# 4-[(*E*)-(2,4-Difluoro­phen­yl)(hydroxy­imino)meth­yl]piperidinium picrate

**DOI:** 10.1107/S1600536809035363

**Published:** 2009-09-05

**Authors:** Jerry P. Jasinski, Ray J. Butcher, H. S. Yathirajan, L. Mallesha, K. N. Mohana

**Affiliations:** a Department of Chemistry, Keene State College, 229 Main Street, Keene, NH 03435-2001, USA; b Department of Chemistry, Howard University, 525 College Street NW, Washington, DC 20059, USA; c Department of Studies in Chemistry, University of Mysore, Manasagangotri, Mysore 570 006, India

## Abstract

The title compound, C_12_H_15_F_2_N_2_O^+^·C_6_H_2_N_3_O_7_^−^, a picrate salt of 4-[(*E*)-(2,4-difluoro­phen­yl)(hydroxy­imino)meth­yl]piper­idine, crystallizes with two independent mol­ecules in a cation–anion pair in the asymmetric unit. In the cation, a methyl group is tris­ubstituted by hydroxy­imino, piperidin-4-yl and 2,4-difluoro­phenyl groups, the latter of which contains an F atom disordered over two positions in the ring [occupancy ratio 0.631 (4):0.369 (4)]. The mean plane of the hydr­oxy group is in a synclinical conformation nearly orthogonal [N—C—C—C = 72.44 (19)°] to the mean plane of the piperidine ring, which adopts a slightly distorted chair conformation. The dihedral angle between the mean plane of the 2,4-difluoro­phenyl and piperidin-4-yl groups is 60.2 (3)°. In the picrate anion, the mean planes of the two *o*-NO_2_ and single *p*-NO_2_ groups adopt twist angles of 5.7 (2), 25.3 (7) and 8.3 (6)°, respectively, with the attached planar benzene ring. The dihedral angle between the mean planes of the benzene ring in the picrate anion and those in the hydroxy­imino, piperidin-4-yl and 2,4-difluoro­phenyl groups in the cation are 84.9 (7), 78.9 (4) and 65.1 (1)°, respectively. Extensive hydrogen-bond inter­actions occur between the cation–anion pair, which help to establish the crystal packing in the unit cell. This includes dual three-center hydrogen bonds with the piperidin-4-yl group, the phenolate and *o*-NO_2_ O atoms of the picrate anion at different positions in the unit cell, which form separate N—H⋯(O,O) bifurcated inter­molecular hydrogen-bond inter­actions. Also, the hydr­oxy group forms a separate hydrogen bond with a nearby piperidin-4-yl N atom, thus providing two groups of hydrogen bonds, which form an infinite two-dimensional network along (011).

## Related literature

(*Z*)-(2,4-Difluoro­phen­yl)(piperidin-4-yl)methanone oxime is an inter­mediate in the preparation of risperidone, an anti­psychotic used to treat schizophrenia, see: Umbricht & Kane, (1995 ▶). For related structures, see: Hu *et al.* (2008 ▶); Jottier *et al.* (1992 ▶); Naveen *et al.* (2007 ▶); Ravikumar & Sridhar (2006 ▶); Yathirajan *et al.* (2005 ▶). For a description of the Cambridge Structural Database, see: Allen (2002 ▶) and for *Mogul*, see: Bruno *et al.* (2004 ▶). For puckering parameters, see: Cremer & Pople (1975 ▶).
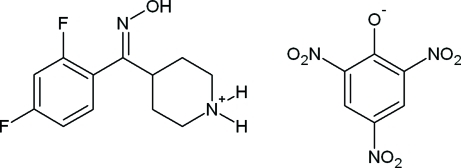


## Experimental

### 

#### Crystal data

C_12_H_15_F_2_N_2_O^+^·C_6_H_2_N_3_O_7_^−^
*M* *_r_* = 469.37
Monoclinic, 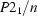

*a* = 6.0926 (4) Å
*b* = 13.5364 (8) Å
*c* = 24.0417 (14) Å
β = 92.671 (6)°
*V* = 1980.63 (19) Å^3^
*Z* = 4
Cu *K*α radiation
μ = 1.20 mm^−1^
*T* = 110 K
0.49 × 0.45 × 0.38 mm

#### Data collection

Oxford Diffraction Xcalibur diffractometer with a Ruby (Gemini Cu) detector
Absorption correction: multi-scan (CrysAlis Pro; Oxford Diffraction, 2009 ▶) *T*_min_ = 0.733, *T*_max_ = 1.000
7628 measured reflections
3910 independent reflections
3475 reflections with *I* > 2σ(*I*)
*R*_int_ = 0.017

#### Refinement

*R*[*F* ^2^ > 2σ(*F* ^2^)] = 0.042
*wR*(*F* ^2^) = 0.115
*S* = 1.05
3910 reflections
303 parameters
H-atom parameters constrained
Δρ_max_ = 0.30 e Å^−3^
Δρ_min_ = −0.30 e Å^−3^

### 

Data collection: *CrysAlis Pro* (Oxford Diffraction, 2009 ▶); cell refinement: *CrysAlis RED* (Oxford Diffraction, 2009 ▶); data reduction: *CrysAlis RED*; program(s) used to solve structure: *SHELXS97* (Sheldrick, 2008 ▶); program(s) used to refine structure: *SHELXL97* (Sheldrick, 2008 ▶); molecular graphics: *SHELXTL* (Sheldrick, 2008 ▶); software used to prepare material for publication: *SHELXTL*.

## Supplementary Material

Crystal structure: contains datablocks global, I. DOI: 10.1107/S1600536809035363/zq2003sup1.cif

Structure factors: contains datablocks I. DOI: 10.1107/S1600536809035363/zq2003Isup2.hkl

Additional supplementary materials:  crystallographic information; 3D view; checkCIF report

## Figures and Tables

**Table 1:** Hydrogen-bond geometry (Å, °)

*D*—H⋯*A*	*D*—H	H⋯*A*	*D*⋯*A*	*D*—H⋯*A*
O1—H1*A*⋯N1^i^	0.84	2.09	2.7980 (18)	141
N2—H2*A*⋯O1*B*	0.92	2.08	2.8005 (16)	134
N2—H2*A*⋯O21*B*	0.92	2.14	2.9580 (17)	148
N2—H2*B*⋯O1*B*^ii^	0.92	1.87	2.7705 (17)	164
N2—H2*B*⋯O62*B*^ii^	0.92	2.56	3.170 (2)	125

Symmetry codes: (i) 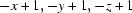; (ii) 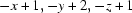.

**Fig. 1.**
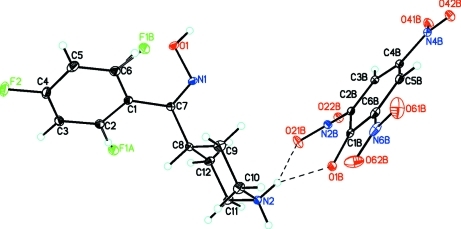
Molecular structure of the title compound, C12H15F2N2O+ C6H2N3O7-, showing the cation-anion unit that comprises the asymmetric unit, the disorder of F1 (F1A & F1B) over two positions (C2 & C6) in the 2,4-difluorophenyl group of the cation, the atom labeling scheme and 50% probability displacement ellipsoids.

**Fig. 2.**
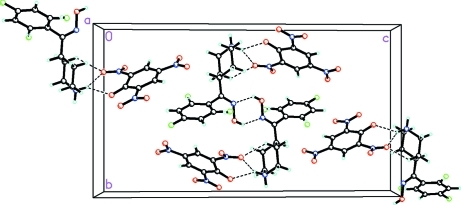
Packing diagram of the title compound, (I), viewed down the a axis. Dashed lines indicate strong N—H···O and weak C—H···O hydrogen bond interactions which produces a two-dimensional network arranged along the (011) plane of the unit cell.

## supplementary crystallographic information

### Comment

The compound, C_12_H_14_F_2_N_2_O, (*Z*)-(2,4-difluorophenyl)(piperidin-4-yl)methanone oxime is an intermediate for the preparation of risperidone. Risperidone contains the functional groups of benzisoxazole and piperidine as part of its molecular structure. Risperidone is an antipsychotic used to treat schizophrenia (Umbricht & Kane, 1995). This drug belongs to a class of anti-psychotic drugs known as neuroleptics and is a strong dopamine antagonist. It has high affinity for D2 dopaminergic receptors.

Related crystal structures of 3-{2-[4-(6-fluoro-1,2-benzisoxazol-3-yl)-1-piperidinyl]ethyl} -2,9-dimethyl-4*H*-pyrido [1,2-*a*]pyrimidin-4-one (ocaperidone) (Jottier *et al.*, 1992), 6-fluoro-3-(4-piperidinio)benz[*d*]isoxazole chloride (Yathirajan *et al.*, 2005), 3-(2-chloroethyl)-2-methyl-4-oxo-6,7,8,9-tetrahydro-4*H*-pyrido [1,2-*a*]pyrimidin-1-ium chloride (Ravikumar & Sridhar, 2006), (2-ethoxyphenyl)[4-(6-fluorobenzo[*d*]isoxazol-3-yl)piperidin-1-yl]methanone (Naveen *et al.*, 2007), (anthracen-9-yl)(piperidin-1-yl)methanone (Hu *et al.*, 2008) have been reported. The present paper reports the interaction of (*Z*)-(2,4-difluorophenyl)(piperidin-4-yl)methanone oxime as an electron donor with picric acid as electron acceptor which resulted in the formation of a charge transfer complex of the title compound, C_18_H_17_F_2_N_5_O_8_, (I).

The title compound, C_12_H_15_F_2_N_2_O^+^ C_6_H_2_N_3_O_7_^-^, a picrate salt of (*E*)-1-(2,4-difluorophenyl)-*N*-hydroxy-1-(piperidin-4-yl) methanimin, crystallizes with two independent molecules in a cation-anion pair in the asymmetric unit. Bond lengths and angles in both the cation and anion can be regarded as normal (Cambridge Structural Database, Version 5.30, February, 2009; Allen, 2002, *Mogul*, Version 1.1.3; Bruno *et al.*, 2004). In the cation, a tri-substituted methyl group contains *N*-hydroxyl, piperidin-4-yl and 2,4-difluorophenyl groups, the latter of which contains a fluoro group (F1A & F1B) disordered over two positions (C2 & C6; Fig. 1), in the phenyl ring, respectively. The mean plane of the hydroxyl group is in a *syn*-clinical conformation (+*sc*, N1—C7—C8—C9 = 72.44 (19)°) nearly orthogonal to the mean plane of the piperidine ring which adopts a slightly distorted chair conformation (Cremer & Pople, 1975) with puckering parameters Q, θ and φ of 0.5781 (17) Å, 1.8 (2)° and 109 (8)°, respectively, For an ideal chair, θ = 0.0°. The dihedral angle between the mean plane of the 2,4-difluorophenyl and piperidin-4-yl groups is 60.2 (3)°. In the picrate anion, the mean planes of the two *o*-NO_2_ and single *p*-NO_2_ groups adopts twist angles of 5.7 (2)°, 25.3 (7)° and 8.3 (6)° with the planar benzene group, respectively. The dihedral angle between the mean planes of the benzene group in the picrate anion and those in the *N*-hydroxyl, piperidin-4-yl and 2,4-difluorophenyl groups in the cation are 76.9 (6)° and 65.0 (7)°, respectively. Extensive hydrogen bond interactions occur between the cation-anion pair which help to establish crystal packing in the unit cell (Fig.2). This includes dual three-center hydrogen bonds with the N2 atom of the piperidin-4-yl group, and the phenolate and *o*-NO_2_ oxygen atoms of the picrate anions at different positions in the unit cell. The H2A and H2B atoms bonded to N2 in the piperidin-4-yl group each forms a separate, N—H···(O,*O*), bifrucated hydrogen bond intermolecular interaction with two different phenolate and *o*-NO_2_ oxygen atoms of the picrate anion at N2–H2A···(O1B,O21B) & N2–H2B···(O1B#2,O62B#2) (#2 = -*x* + 1, -*y* + 2, -*z* + 1; Table 1). Also, the hydroxyl group forms a separate hydrogen bond with a nearby N1 amino group (O1–H1A···N1; -*x* + 1, -*y* + 1, -*z* + 1). These two groups of hydrogen bonds form an infinite 2-D network along the (011) plane of the unit cell (Fig. 2). In addition, a weak C–H···O π-ring intermolecular hydrogen bond interaction [C3B—H3BA···*Cg*2; H3BA···*Cg*2 = 2.95 Å, C3B···*Cg*2 = 3.75 (4) Å, C3B—H3BA···*Cg*2 = 143°; *Cg*2 = C1–C6)] is observed which helps to stablize crystal packing.

### Experimental

The title compound was synthesized by adding a saturated solution of picric acid (0.92 g, 2 mmol) in 10 ml me thanol to a solution of (*Z*)-(2,4-difluorophenyl)(piperidin-4-yl)methanone oxime (0.49 g, 2 mmol) in 10 ml me thanol. A yellow color developed and the solution was allowed to evaporate slowly at room temperature. The yellow colored complex formed was filtered off, washed several times with diethyl ether and then dried over CaCl_2_ (yield: 64.5%). X-ray quality crystals were grown from a methanol solution. The melting range was found to be 459–462 K. Analysis found (calculated) for C_18_H_17_F_2_N_5_O_8_ (%): C: 46.07 (46.06), H: 3.85 (3.65), N: 14.95 (14.92).

### Refinement

The hydroxyl hydrogen, H1A, was obtained from a difference fourier map. All of the remaining H atoms were placed in their calculated positions and then refined using riding models with N—H = 0.92 Å, C—H = 0.95–1.00 Å, and with *U_i_*_so_(H) = 1.18–1.218*U*_eq_(C,N).

### Crystal data

**Table:**

C_12_H_15_F_2_N_2_O^+^·C_6_H_2_N_3_O_7_^−^	*F*(000) = 968
*M**_r_* = 469.37	*D*_x_ = 1.574 Mg m^−^^3^
Monoclinic, *P*2_1_/*n*	Cu *K*α radiation, λ = 1.54184 Å
Hall symbol: -P 2yn	Cell parameters from 4482 reflections
*a* = 6.0926 (4) Å	θ = 4.9–74.1°
*b* = 13.5364 (8) Å	µ = 1.20 mm^−^^1^
*c* = 24.0417 (14) Å	*T* = 110 K
β = 92.671 (6)°	Chunk, pale yellow
*V* = 1980.63 (19) Å^3^	0.49 × 0.45 × 0.38 mm
*Z* = 4	

### Data collection

**Table:**

Oxford Diffraction Xcalibur diffractometer with a Ruby (Gemini Cu) detector	3910 independent reflections
Radiation source: Enhance (Cu) X-ray Source	3475 reflections with *I* > 2σ(*I*)
graphite	*R*_int_ = 0.017
Detector resolution: 10.5081 pixels mm^-1^	θ_max_ = 74.1°, θ_min_ = 4.9°
ω scans	*h* = −6→7
Absorption correction: multi-scan (*CrysAlis PRO*; Oxford Diffraction, 2009)	*k* = −11→16
*T*_min_ = 0.734, *T*_max_ = 1.000	*l* = −29→29
7628 measured reflections	

### Refinement

**Table:**

Refinement on *F*^2^	Primary atom site location: structure-invariant direct methods
Least-squares matrix: full	Secondary atom site location: difference Fourier map
*R*[*F*^2^ > 2σ(*F*^2^)] = 0.042	Hydrogen site location: inferred from neighbouring sites
*wR*(*F*^2^) = 0.115	H-atom parameters constrained
*S* = 1.05	*w* = 1/[σ^2^(*F*_o_^2^) + (0.0619*P*)^2^ + 1.0569*P*] where *P* = (*F*_o_^2^ + 2*F*_c_^2^)/3
3910 reflections	(Δ/σ)_max_ < 0.001
303 parameters	Δρ_max_ = 0.30 e Å^−^^3^
0 restraints	Δρ_min_ = −0.30 e Å^−^^3^

### Special details

**Table:**

Experimental. CrysAlisPro, Oxford Diffraction Ltd., Version 1.171.33.34d (release 27-02-2009 CrysAlis171 .NET) (compiled Feb 27 2009,15:38:38) Empirical absorption correction using spherical harmonics, implemented in SCALE3 ABSPACK scaling algorithm.
Geometry. All e.s.d.'s (except the e.s.d. in the dihedral angle between two l.s. planes) are estimated using the full covariance matrix. The cell e.s.d.'s are taken into account individually in the estimation of e.s.d.'s in distances, angles and torsion angles; correlations between e.s.d.'s in cell parameters are only used when they are defined by crystal symmetry. An approximate (isotropic) treatment of cell e.s.d.'s is used for estimating e.s.d.'s involving l.s. planes.
Refinement. Refinement of *F*^2^ against ALL reflections. The weighted *R*-factor *wR* and goodness of fit *S* are based on *F*^2^, conventional *R*-factors *R* are based on *F*, with *F* set to zero for negative *F*^2^. The threshold expression of *F*^2^ > σ(*F*^2^) is used only for calculating *R*-factors(gt) *etc*. and is not relevant to the choice of reflections for refinement. *R*-factors based on *F*^2^ are statistically about twice as large as those based on *F*, and *R*- factors based on ALL data will be even larger.

### Fractional atomic coordinates and isotropic or equivalent isotropic displacement parameters (Å^2^)

**Table:**

	*x*	*y*	*z*	*U*_iso_*/*U*_eq_	Occ. (<1)
F1A	0.4117 (3)	0.58470 (13)	0.70084 (6)	0.0326 (4)	0.631 (4)
F1B	−0.0826 (5)	0.4942 (2)	0.55863 (11)	0.0326 (4)	0.37
F2	−0.2362 (2)	0.41781 (9)	0.74432 (5)	0.0519 (4)	
O1	0.3558 (2)	0.44002 (9)	0.54602 (6)	0.0389 (3)	
H1A	0.4171	0.4159	0.5185	0.058*	
N1	0.3999 (3)	0.54236 (11)	0.54919 (6)	0.0309 (3)	
N2	0.5153 (2)	0.88427 (9)	0.55578 (5)	0.0199 (3)	
H2A	0.5653	0.8665	0.5217	0.024*	
H2B	0.5390	0.9509	0.5604	0.024*	
C1	0.1660 (3)	0.53694 (11)	0.62919 (6)	0.0214 (3)	
C2	0.2218 (3)	0.53902 (12)	0.68601 (7)	0.0249 (3)	
H2	0.3559	0.5695	0.6982	0.030*	0.369 (4)
C3	0.0908 (3)	0.49879 (12)	0.72519 (7)	0.0299 (4)	
H3A	0.1331	0.4999	0.7637	0.036*	
C4	−0.1038 (3)	0.45693 (13)	0.70620 (8)	0.0332 (4)	
C5	−0.1698 (3)	0.45176 (13)	0.65070 (8)	0.0321 (4)	
H5A	−0.3052	0.4218	0.6390	0.039*	
C6	−0.0313 (3)	0.49188 (12)	0.61243 (7)	0.0261 (3)	
H6	−0.0723	0.4885	0.5739	0.031*	0.631 (4)
C7	0.3115 (3)	0.58538 (12)	0.58942 (6)	0.0221 (3)	
C8	0.3575 (2)	0.69523 (11)	0.59583 (6)	0.0206 (3)	
H8A	0.3034	0.7169	0.6325	0.025*	
C9	0.2332 (3)	0.75380 (13)	0.55000 (7)	0.0273 (4)	
H9A	0.2810	0.7318	0.5132	0.033*	
H9B	0.0738	0.7405	0.5516	0.033*	
C10	0.2742 (3)	0.86397 (13)	0.55644 (8)	0.0286 (4)	
H10A	0.1964	0.9001	0.5256	0.034*	
H10B	0.2167	0.8873	0.5920	0.034*	
C11	0.6425 (3)	0.82942 (11)	0.60045 (7)	0.0237 (3)	
H11A	0.5979	0.8524	0.6374	0.028*	
H11B	0.8012	0.8432	0.5977	0.028*	
C12	0.6024 (3)	0.71882 (11)	0.59519 (7)	0.0230 (3)	
H12A	0.6812	0.6842	0.6264	0.028*	
H12B	0.6612	0.6947	0.5600	0.028*	
O1B	0.48994 (19)	0.91121 (8)	0.44016 (5)	0.0257 (3)	
O21B	0.7803 (2)	0.77495 (10)	0.47615 (5)	0.0327 (3)	
O22B	1.01401 (18)	0.74502 (9)	0.41357 (5)	0.0262 (3)	
O41B	0.7531 (3)	0.69201 (10)	0.22667 (5)	0.0441 (4)	
O42B	0.4368 (3)	0.74855 (11)	0.19724 (5)	0.0426 (4)	
O61B	−0.0029 (2)	0.93104 (14)	0.31606 (7)	0.0563 (5)	
O62B	0.1039 (2)	0.97294 (11)	0.39934 (8)	0.0495 (4)	
N2B	0.8313 (2)	0.77022 (10)	0.42725 (5)	0.0207 (3)	
N4B	0.5790 (3)	0.73383 (11)	0.23427 (6)	0.0322 (4)	
N6B	0.1329 (2)	0.93020 (11)	0.35541 (7)	0.0327 (4)	
C1B	0.4929 (2)	0.86302 (11)	0.39577 (6)	0.0197 (3)	
C2B	0.6651 (2)	0.79351 (11)	0.38377 (6)	0.0191 (3)	
C3B	0.6944 (3)	0.75188 (11)	0.33251 (6)	0.0219 (3)	
H3BA	0.8174	0.7107	0.3266	0.026*	
C4B	0.5401 (3)	0.77135 (12)	0.28969 (6)	0.0249 (3)	
C5B	0.3567 (3)	0.82860 (12)	0.29821 (7)	0.0268 (4)	
H5BA	0.2481	0.8377	0.2690	0.032*	
C6B	0.3327 (3)	0.87222 (11)	0.34922 (7)	0.0241 (3)	

### Atomic displacement parameters (Å^2^)

**Table:**

	*U*^11^	*U*^22^	*U*^33^	*U*^12^	*U*^13^	*U*^23^
F1A	0.0361 (8)	0.0377 (8)	0.0237 (7)	−0.0054 (6)	−0.0035 (5)	−0.0009 (6)
F1B	0.0361 (8)	0.0377 (8)	0.0237 (7)	−0.0054 (6)	−0.0035 (5)	−0.0009 (6)
F2	0.0645 (8)	0.0379 (6)	0.0566 (8)	−0.0099 (6)	0.0386 (6)	0.0049 (5)
O1	0.0580 (8)	0.0199 (6)	0.0410 (7)	−0.0143 (6)	0.0276 (6)	−0.0133 (5)
N1	0.0441 (8)	0.0187 (7)	0.0313 (7)	−0.0118 (6)	0.0156 (6)	−0.0087 (6)
N2	0.0251 (6)	0.0157 (6)	0.0189 (6)	0.0022 (5)	0.0021 (5)	0.0010 (5)
C1	0.0263 (8)	0.0147 (7)	0.0235 (8)	0.0008 (6)	0.0064 (6)	−0.0009 (6)
C2	0.0305 (8)	0.0187 (7)	0.0259 (8)	0.0016 (6)	0.0055 (6)	−0.0020 (6)
C3	0.0466 (10)	0.0196 (8)	0.0243 (8)	0.0054 (7)	0.0104 (7)	0.0013 (6)
C4	0.0428 (10)	0.0196 (8)	0.0390 (10)	0.0010 (7)	0.0229 (8)	0.0028 (7)
C5	0.0254 (8)	0.0229 (8)	0.0488 (11)	−0.0027 (7)	0.0106 (7)	−0.0037 (7)
C6	0.0282 (8)	0.0209 (8)	0.0293 (8)	0.0014 (6)	0.0040 (7)	−0.0026 (6)
C7	0.0259 (7)	0.0205 (8)	0.0203 (7)	−0.0040 (6)	0.0032 (6)	−0.0024 (6)
C8	0.0265 (8)	0.0183 (7)	0.0175 (7)	−0.0021 (6)	0.0050 (6)	−0.0023 (6)
C9	0.0177 (7)	0.0291 (9)	0.0347 (9)	0.0012 (6)	−0.0053 (6)	−0.0001 (7)
C10	0.0221 (8)	0.0257 (8)	0.0378 (9)	0.0083 (6)	0.0004 (7)	0.0045 (7)
C11	0.0259 (8)	0.0185 (7)	0.0259 (8)	−0.0014 (6)	−0.0080 (6)	0.0022 (6)
C12	0.0238 (8)	0.0164 (7)	0.0277 (8)	−0.0002 (6)	−0.0077 (6)	0.0025 (6)
O1B	0.0371 (6)	0.0189 (5)	0.0212 (5)	0.0061 (5)	0.0042 (5)	0.0021 (4)
O21B	0.0333 (6)	0.0485 (8)	0.0163 (5)	0.0145 (6)	0.0005 (5)	−0.0001 (5)
O22B	0.0217 (5)	0.0304 (6)	0.0266 (6)	0.0043 (5)	0.0024 (4)	−0.0007 (5)
O41B	0.0744 (10)	0.0356 (7)	0.0227 (6)	0.0128 (7)	0.0063 (6)	−0.0007 (5)
O42B	0.0634 (9)	0.0424 (8)	0.0206 (6)	−0.0175 (7)	−0.0141 (6)	0.0038 (5)
O61B	0.0276 (7)	0.0832 (12)	0.0574 (9)	0.0107 (7)	−0.0072 (6)	0.0273 (9)
O62B	0.0328 (7)	0.0341 (8)	0.0810 (11)	0.0108 (6)	−0.0029 (7)	−0.0201 (8)
N2B	0.0226 (6)	0.0205 (6)	0.0189 (6)	0.0015 (5)	0.0000 (5)	−0.0005 (5)
N4B	0.0563 (10)	0.0218 (7)	0.0179 (7)	−0.0109 (7)	−0.0037 (6)	0.0033 (5)
N6B	0.0222 (7)	0.0233 (7)	0.0525 (10)	−0.0023 (6)	−0.0006 (6)	0.0156 (7)
C1B	0.0239 (7)	0.0154 (7)	0.0202 (7)	−0.0029 (6)	0.0028 (6)	0.0041 (5)
C2B	0.0213 (7)	0.0175 (7)	0.0183 (7)	−0.0028 (6)	−0.0002 (5)	0.0029 (6)
C3B	0.0289 (8)	0.0169 (7)	0.0199 (7)	−0.0026 (6)	0.0015 (6)	0.0008 (6)
C4B	0.0373 (9)	0.0192 (7)	0.0180 (7)	−0.0076 (7)	−0.0019 (6)	0.0020 (6)
C5B	0.0300 (8)	0.0243 (8)	0.0253 (8)	−0.0098 (6)	−0.0086 (6)	0.0092 (6)
C6B	0.0216 (7)	0.0188 (7)	0.0318 (8)	−0.0019 (6)	−0.0007 (6)	0.0081 (6)

### Geometric parameters (Å, °)

**Table:**

F2—C4	1.3563 (19)	C10—H10A	0.9900
O1—N1	1.4126 (18)	C10—H10B	0.9900
O1—H1A	0.8400	C11—C12	1.521 (2)
N1—C7	1.270 (2)	C11—H11A	0.9900
N2—C11	1.4926 (19)	C11—H11B	0.9900
N2—C10	1.495 (2)	C12—H12A	0.9900
N2—H2A	0.9200	C12—H12B	0.9900
N2—H2B	0.9200	O1B—C1B	1.2517 (19)
C1—C6	1.391 (2)	O21B—N2B	1.2317 (17)
C1—C2	1.393 (2)	O22B—N2B	1.2240 (17)
C1—C7	1.486 (2)	O41B—N4B	1.224 (2)
C2—C3	1.375 (2)	O42B—N4B	1.229 (2)
C2—H2	0.9500	O61B—N6B	1.227 (2)
C3—C4	1.373 (3)	O62B—N6B	1.224 (2)
C3—H3A	0.9500	N2B—C2B	1.4556 (19)
C4—C5	1.377 (3)	N4B—C4B	1.456 (2)
C5—C6	1.388 (2)	N6B—C6B	1.462 (2)
C5—H5A	0.9500	C1B—C2B	1.448 (2)
C6—H6	0.9500	C1B—C6B	1.456 (2)
C7—C8	1.520 (2)	C2B—C3B	1.374 (2)
C8—C12	1.526 (2)	C3B—C4B	1.387 (2)
C8—C9	1.529 (2)	C3B—H3BA	0.9500
C8—H8A	1.0000	C4B—C5B	1.383 (3)
C9—C10	1.519 (2)	C5B—C6B	1.375 (2)
C9—H9A	0.9900	C5B—H5BA	0.9500
C9—H9B	0.9900		
			
N1—O1—H1A	109.5	C9—C10—H10A	109.7
C7—N1—O1	113.86 (13)	N2—C10—H10B	109.7
C11—N2—C10	112.24 (12)	C9—C10—H10B	109.7
C11—N2—H2A	109.2	H10A—C10—H10B	108.2
C10—N2—H2A	109.2	N2—C11—C12	110.71 (12)
C11—N2—H2B	109.2	N2—C11—H11A	109.5
C10—N2—H2B	109.2	C12—C11—H11A	109.5
H2A—N2—H2B	107.9	N2—C11—H11B	109.5
C6—C1—C2	117.46 (14)	C12—C11—H11B	109.5
C6—C1—C7	122.72 (14)	H11A—C11—H11B	108.1
C2—C1—C7	119.78 (14)	C11—C12—C8	111.00 (13)
C3—C2—C1	122.77 (16)	C11—C12—H12A	109.4
C3—C2—H2	118.6	C8—C12—H12A	109.4
C1—C2—H2	118.6	C11—C12—H12B	109.4
C4—C3—C2	117.09 (16)	C8—C12—H12B	109.4
C4—C3—H3A	121.5	H12A—C12—H12B	108.0
C2—C3—H3A	121.5	O22B—N2B—O21B	122.94 (13)
F2—C4—C3	117.88 (17)	O22B—N2B—C2B	118.59 (12)
F2—C4—C5	118.62 (17)	O21B—N2B—C2B	118.45 (13)
C3—C4—C5	123.49 (16)	O41B—N4B—O42B	123.60 (15)
C4—C5—C6	117.61 (16)	O41B—N4B—C4B	118.50 (14)
C4—C5—H5A	121.2	O42B—N4B—C4B	117.86 (17)
C6—C5—H5A	121.2	O62B—N6B—O61B	122.80 (16)
C5—C6—C1	121.57 (16)	O62B—N6B—C6B	119.71 (15)
C5—C6—H6	119.2	O61B—N6B—C6B	117.45 (17)
C1—C6—H6	119.2	O1B—C1B—C2B	123.23 (14)
N1—C7—C1	125.05 (14)	O1B—C1B—C6B	125.10 (14)
N1—C7—C8	116.31 (14)	C2B—C1B—C6B	111.61 (13)
C1—C7—C8	118.63 (13)	C3B—C2B—C1B	124.81 (14)
C7—C8—C12	112.30 (13)	C3B—C2B—N2B	116.10 (14)
C7—C8—C9	110.57 (13)	C1B—C2B—N2B	118.93 (13)
C12—C8—C9	109.62 (12)	C2B—C3B—C4B	118.41 (15)
C7—C8—H8A	108.1	C2B—C3B—H3BA	120.8
C12—C8—H8A	108.1	C4B—C3B—H3BA	120.8
C9—C8—H8A	108.1	C5B—C4B—C3B	121.40 (15)
C10—C9—C8	111.22 (13)	C5B—C4B—N4B	119.87 (15)
C10—C9—H9A	109.4	C3B—C4B—N4B	118.67 (16)
C8—C9—H9A	109.4	C6B—C5B—C4B	119.53 (15)
C10—C9—H9B	109.4	C6B—C5B—H5BA	120.2
C8—C9—H9B	109.4	C4B—C5B—H5BA	120.2
H9A—C9—H9B	108.0	C5B—C6B—C1B	123.61 (15)
N2—C10—C9	109.65 (13)	C5B—C6B—N6B	116.43 (15)
N2—C10—H10A	109.7	C1B—C6B—N6B	119.96 (15)
			
C6—C1—C2—C3	0.0 (2)	O1B—C1B—C2B—C3B	−168.56 (15)
C7—C1—C2—C3	177.62 (15)	C6B—C1B—C2B—C3B	9.0 (2)
C1—C2—C3—C4	−1.2 (2)	O1B—C1B—C2B—N2B	6.6 (2)
C2—C3—C4—F2	−179.33 (15)	C6B—C1B—C2B—N2B	−175.87 (12)
C2—C3—C4—C5	1.4 (3)	O22B—N2B—C2B—C3B	23.4 (2)
F2—C4—C5—C6	−179.72 (15)	O21B—N2B—C2B—C3B	−155.21 (14)
C3—C4—C5—C6	−0.5 (3)	O22B—N2B—C2B—C1B	−152.14 (14)
C4—C5—C6—C1	−0.8 (2)	O21B—N2B—C2B—C1B	29.2 (2)
C2—C1—C6—C5	1.0 (2)	C1B—C2B—C3B—C4B	−5.0 (2)
C7—C1—C6—C5	−176.53 (15)	N2B—C2B—C3B—C4B	179.71 (13)
O1—N1—C7—C1	−2.5 (3)	C2B—C3B—C4B—C5B	−2.0 (2)
O1—N1—C7—C8	178.61 (14)	C2B—C3B—C4B—N4B	175.31 (14)
C6—C1—C7—N1	−60.4 (2)	O41B—N4B—C4B—C5B	172.66 (15)
C2—C1—C7—N1	122.18 (19)	O42B—N4B—C4B—C5B	−5.3 (2)
C6—C1—C7—C8	118.49 (17)	O41B—N4B—C4B—C3B	−4.7 (2)
C2—C1—C7—C8	−59.0 (2)	O42B—N4B—C4B—C3B	177.34 (15)
N1—C7—C8—C12	−50.3 (2)	C3B—C4B—C5B—C6B	4.0 (2)
C1—C7—C8—C12	130.70 (15)	N4B—C4B—C5B—C6B	−173.31 (14)
N1—C7—C8—C9	72.44 (19)	C4B—C5B—C6B—C1B	0.9 (2)
C1—C7—C8—C9	−106.51 (16)	C4B—C5B—C6B—N6B	−178.96 (14)
C7—C8—C9—C10	178.97 (13)	O1B—C1B—C6B—C5B	170.67 (15)
C12—C8—C9—C10	−56.69 (17)	C2B—C1B—C6B—C5B	−6.8 (2)
C11—N2—C10—C9	−57.77 (17)	O1B—C1B—C6B—N6B	−9.5 (2)
C8—C9—C10—N2	57.41 (18)	C2B—C1B—C6B—N6B	173.04 (13)
C10—N2—C11—C12	57.29 (17)	O62B—N6B—C6B—C5B	−178.23 (15)
N2—C11—C12—C8	−55.84 (17)	O61B—N6B—C6B—C5B	3.9 (2)
C7—C8—C12—C11	178.80 (13)	O62B—N6B—C6B—C1B	1.9 (2)
C9—C8—C12—C11	55.48 (17)	O61B—N6B—C6B—C1B	−175.92 (15)

### Hydrogen-bond geometry (Å, °)

**Table:**

*D*—H···*A*	*D*—H	H···*A*	*D*···*A*	*D*—H···*A*
O1—H1A···N1^i^	0.84	2.09	2.7980 (18)	141
N2—H2A···O1B	0.92	2.08	2.8005 (16)	134
N2—H2A···O21B	0.92	2.14	2.9580 (17)	148
N2—H2B···O1B^ii^	0.92	1.87	2.7705 (17)	164
N2—H2B···O62B^ii^	0.92	2.56	3.170 (2)	125

Symmetry codes: (i) −*x*+1, −*y*+1, −*z*+1; (ii) −*x*+1, −*y*+2, −*z*+1.
